# Supplementary material for: IGF-1R/mTOR Targeted Therapy for Ewing Sarcoma: A Meta-Analysis of Five IGF-1R-Related Trials Matched to Proteomic and Radiologic Predictive Biomarkers
Source: Cancers (Basel). 2020 Jul 2;12(7):1768. doi: 10.3390/cancers12071768 (PMC7408058; doi:10.3390/cancers12071768)
Supplement: Supplementary file 1 [file cancers-12-01768-s001.pdf]

cancers

MDPI

Supplementary Materials

IGF-1R/mTOR Targeted Therapy for Ewing Sarcoma: A Meta-Analysis of Five IGF-1R-Related Trials Matched to Proteomic and Radiologic Predictive Biomarkers

Hesham M. Amin [1,†], Ajaykumar C. Morani [2,†], Najat C. Daw [3], Salah-Eddine Lamhamedi-Cherradi [4], Vivek Subbiah [5], Brian A. Menegaz [6], Deeksha Vishwamitra [1], Ghazaleh Eskandari [1], Bhawana George [1], Robert S. Benjamin [4], Shreyaskumar Patel [4], Juhee Song [7], Alexander J. Lazar [8], Wei-Lien Wang [8], Razelle Kurzrock [9], Alberto Pappo [10], Peter M. Anderson [11], Gary K. Schwartz [12], Dejka Araujo [4], Branko Cuglievan [3], Ravin Ratan [4], David McCall [3], Sana Mohiuddin [3], John A. Livingston [4], Eric R. Molina [6], Aung Naing [5,‡] and Joseph A. Ludwig [4,‡,*]

Methods and Materials

Human Subjects

The study was not powered to detect whether IGF-1R Abs from different companies were more or less effective than others included in the study, and we made no attempt to compare them. PET/CTs were obtained using the guidelines set forth in each of the five clinical trials. Prior to the current analysis, each study's research findings were published, and we refer you to those publications for additional information regarding drug safety, clinical outcome, and other correlative analysis.

Statistical Analyses

Progression free survival (PFS) was defined as a time interval from initiation of treatment to progression for each treatment record. Overall survival (OS) was defined as a time interval from initiation of treatment to death or last follow-up.

Three statistical models were used to evaluate PFS: Method A (detailed in the text of the publication), and Methods B and C, which included the 5 patients who had enrolled in both the IGF-1R and IGF-1R/mTORi studies. Method B evaluated all 56 patients but excluded the 2nd treatment record for 5 patients that enrolled on both studies. Method C assessed all 61 treatment records, considering treatment as a time varying covariate. In the time varying covariate analysis, the PFS for those who received 2 treatments were divided into 3 intervals: (a) initiation of 1st treatment to the end of 1st treatment, (b) gap between 1st treatment and the 2nd treatment, (c) initiation of 2nd treatment to the end of 2nd treatment. The progression status at the end of each interval was used, and if the progression status was unknown in the gap between the 1st and the 2nd treatments, the PFS was censored.

Three different analyses were performed to evaluate OS: Method A included 51 patients who received only 1 treatment; Method B used 56 treatment records, censoring those who received 2 treatments at the initiation of 2nd treatment, and Method C used the time varying covariate approach. In this latter approach, the survival for those who received 2 treatments was calculated by dividing the treatment period into 3 intervals: (a) initiation of 1st treatment to the end of 1st treatment, (b) gap between 1st treatment and the 2nd treatment, and (c) initiation of 2nd treatment to death or last follow-up. Survival status at the end of each interval was used. From the initiation of 1st treatment to the end of 1st treatment and gap between 1st treatment and the 2nd treatment, survival status was alive and OS was censored. For the comparison of anti-tumor effects and clinical benefit between 2 treatments, the same method A and method B were implemented. Method C evaluated 61 records assuming

independence for 2 treatment records from the same patient. A p-value of less than 0.05 indicated a statistical significance. SAS 9.4 (SAS Institute INC, Cary, NC, USA) was used for data analysis.

Immunohistochemical Staining

The intensity of protein expression was scored as: 0, negative; +1, weak; +2, intermediate; +3, strong. A protein was considered positive when ≥ 20% of the cells had a score of +1 or more. Photomicrographs were captured using a Nikon Microphot FXA microscope (Nikon Instruments; Melville, NJ, USA), an Olympus DP70 camera (Olympus America; Jupiter, FL, USA), and the QCapture Suite PLUS software (QImaging; Surrey, British Columbia, CA, USA). Slides were then developed with 3,3′-diaminodbenzidine tetrahydrochloride substrate (DAB) that included horseradish peroxidase enzyme. Hematoxylin was used for counter staining.

Immunofluorescence Staining

A673 Ewing sarcoma primary cells treated during 2 h with IGF-1 ligand (50 ng/ml, R&D Biosystem, Minneapolis, MN, USA) or not were fixed with 4% paraformaldehyde (in 1X PBS) for 10 min. After washing with PBS, cells were permeabilized and blocked with superblock buffer (Thermo Fisher Scientific, Sugar Land, TX, USA) for 1 hour. Cells were incubated with anti pIGF-1R-Y1161 antibody (Abcam, ab39398) diluted (1:100) in superblock buffer at 4C overnight in a humidity chamber. The primary antibody was removed, cells washed, then incubated with Alexa Fluor 568-conjugated secondary antibody (Thermo Fisher Scientific) for 1 h at room temperature. The nuclei were visualized using Hoechst (Thermo Fisher Scientific) and the immunofluorescence was visualized using the Nikon A1-Rsi confocal microscope (Nikon, Houston, TX, USA). The fluorescence labeled pIGF-1R-Y1161 protein in both nuclei and cytosol regions was quantified using the Imaris software (Version 9.2; Bitplane, Saint Paul, MN, USA) and its Cell module to define the segmentation by permitting the recognition of pIGF-1R-Y1161 fluorescence in both nuclear and cytosolic regions.

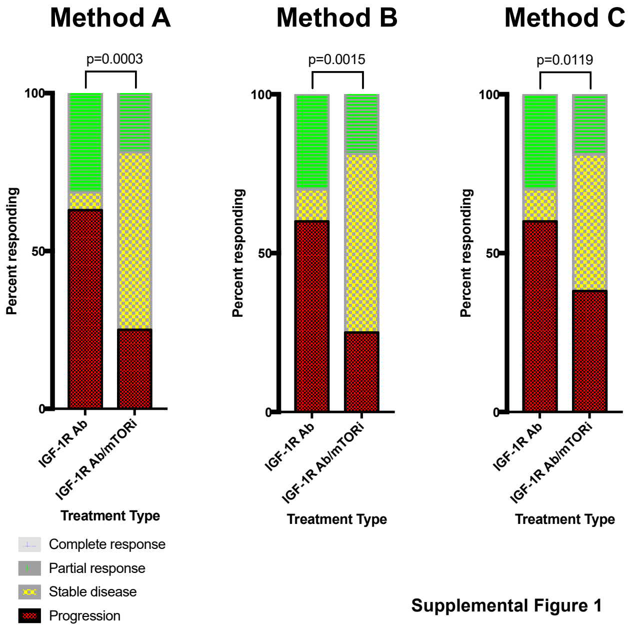

Figure S1. Compared to single-agent IGF-1R Abs, the IGF-1R/mTOR-based combination leads to better anti-tumor effects and superior PFS irrespective of the statistical methods used. Method A assessed 51 patients that received an IGF-1R Ab or IGF-1R/mTOR combination but excluded any patient that received both modalities. Method B evaluated 56 patients for their first treatment but excluded the second treatment record of any patient that received an IGF-1R Abs followed by an IGF-1R/mTOR combination. Method C includes all data from the 56 patients, evaluating 61 treatment records as if they are independent events.

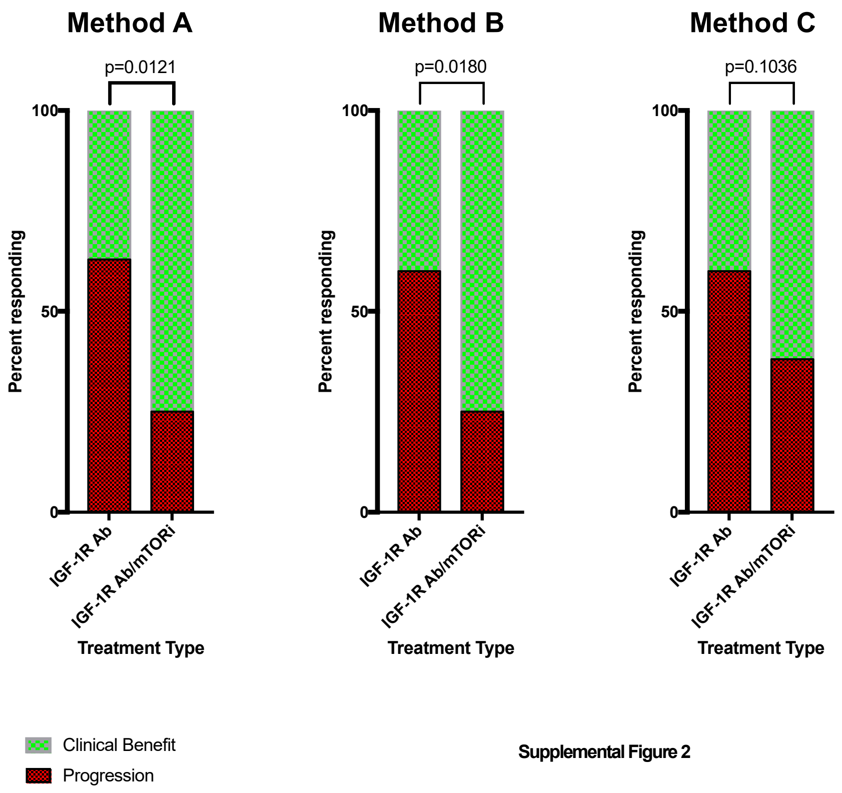

Figure S2. Compared to single-agent IGF-1R Abs, the IGF-1R/mTOR-based combination leads to superior clinical benefit (i.e. stable disease, partial response, or complete response) irrespective of the statistical methods used. Method A assessed 51 patients that received an IGF-1R Ab or IGF-1R/mTOR combination but excluded any patient that received both modalities. Method B evaluated 56 patients for their first treatment but excluded the second treatment record of any patient that received an IGF-1R Abs followed by an IGF-1R/mTOR combination. Method C includes all data from the 56 patients, evaluating 61 treatment records as if they are independent events.

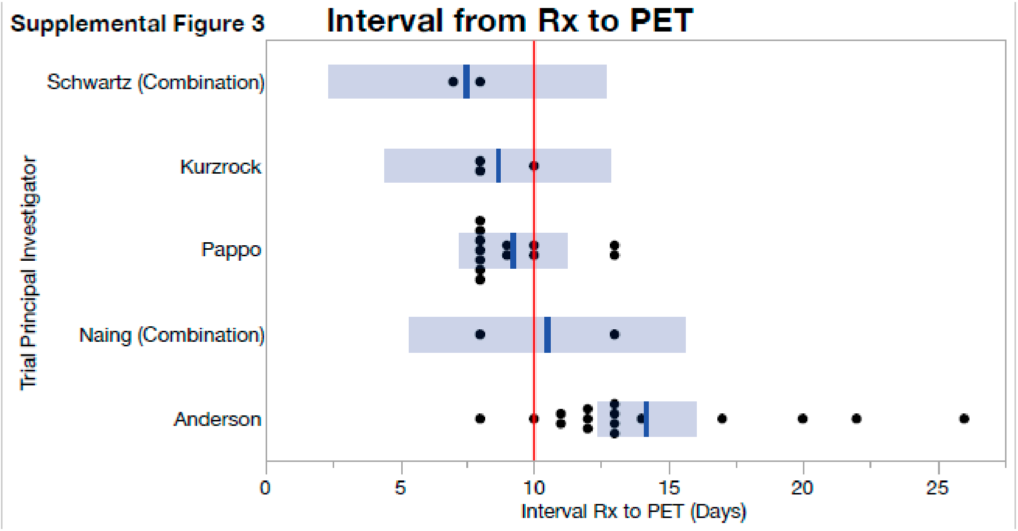

Figure S3. The interval between the first dose of an IGF-1R Ab +/− mTORi until the research-only PET/CT was conducted, separated by each trial's nationwide principal investigator. Each dot represents a single patient. The red vertical line indicates the median time to PET/CT imaging for all studies.

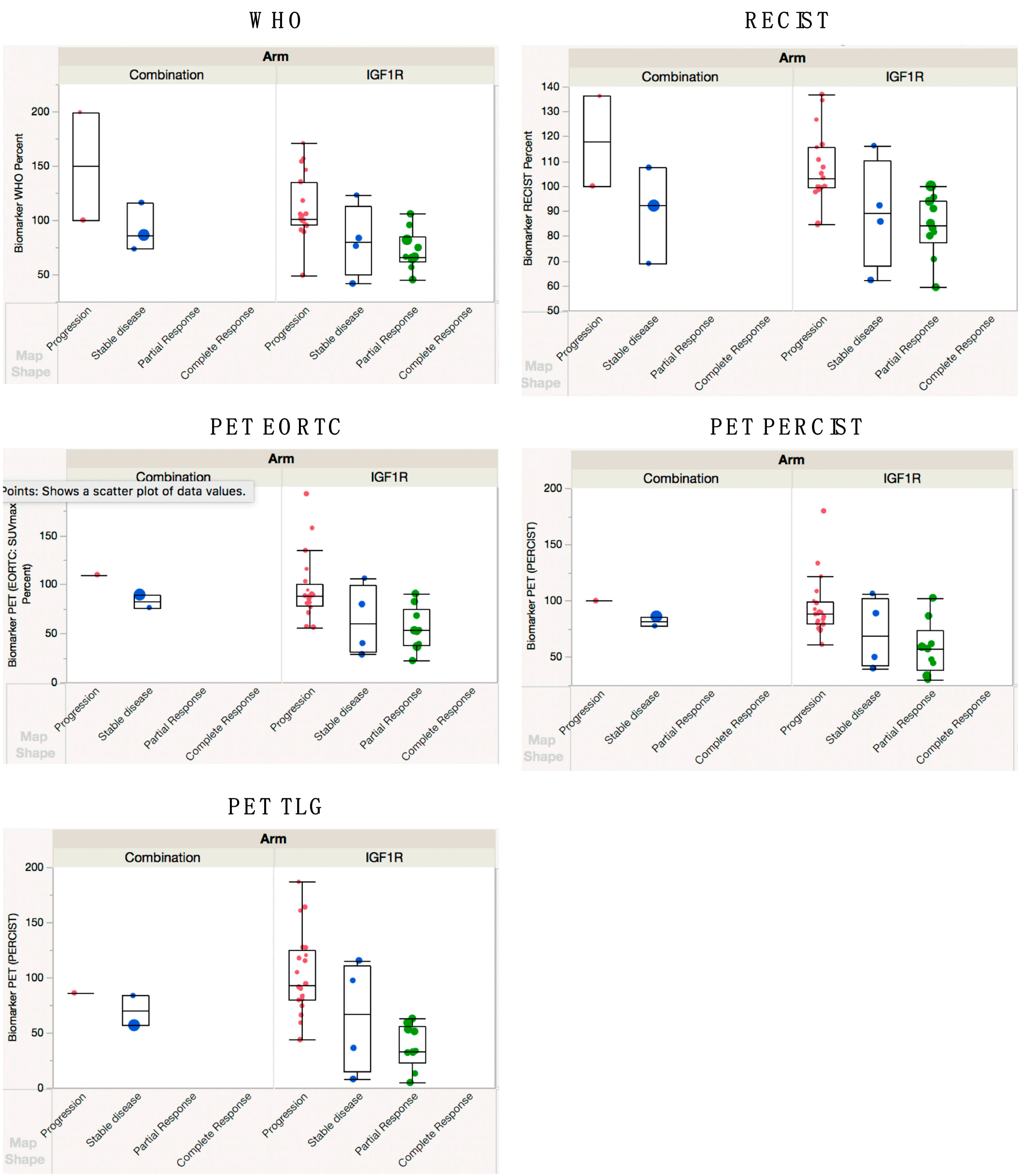

Figure S4. Early response criterion from anatomical and radiological imaging. Dot plot of each individual patient's response from the early research image obtained between days 7–14, grouped by the image response metric and stratified by the treatment arm (e.g., dual IGF- 1R/mTOR inhibitors vs. IGF-1R Ab). Though all 56 patients were included in this analysis, not all patients underwent early imaging to investigate the role of CT or PET as a predictive biomarker. Box plot overlay indicates quantiles and each circle diameter represents the duration of PFS.

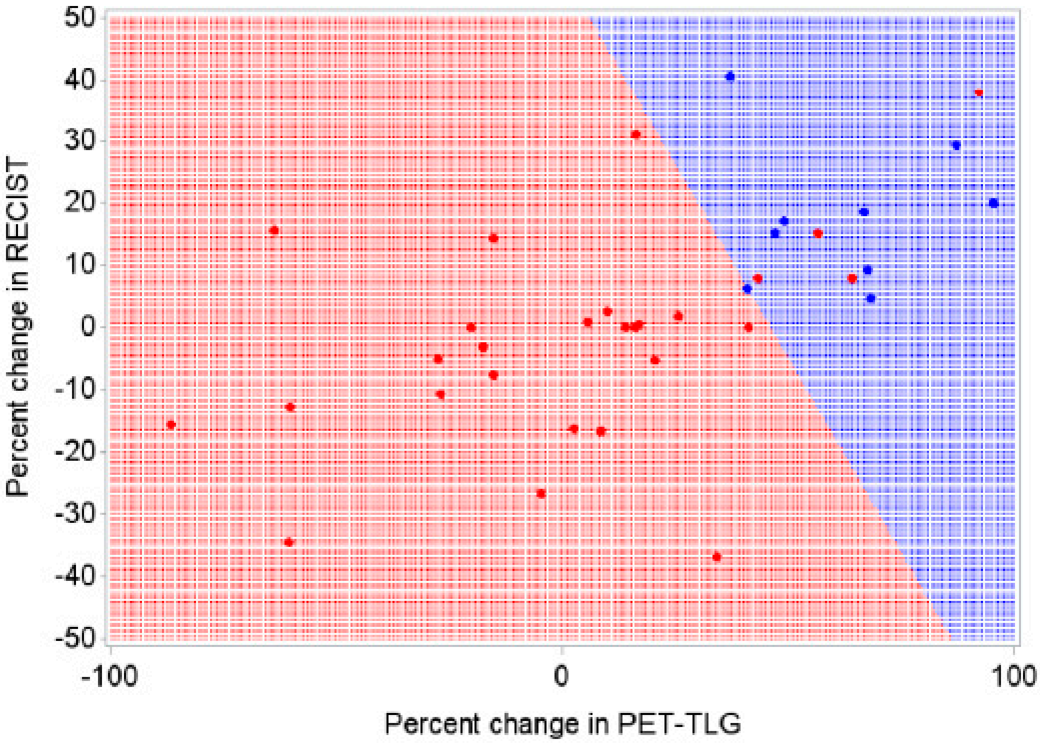

Figure S5. Multivariable logistic regression model using percent tumor response from early day 7–14 imaging to predict best clinical response on therapy. RESIST criteria were used to quantify tumor size; TLG was used to measure tumor metabolic activity. No response (red dots); response (blue dots).

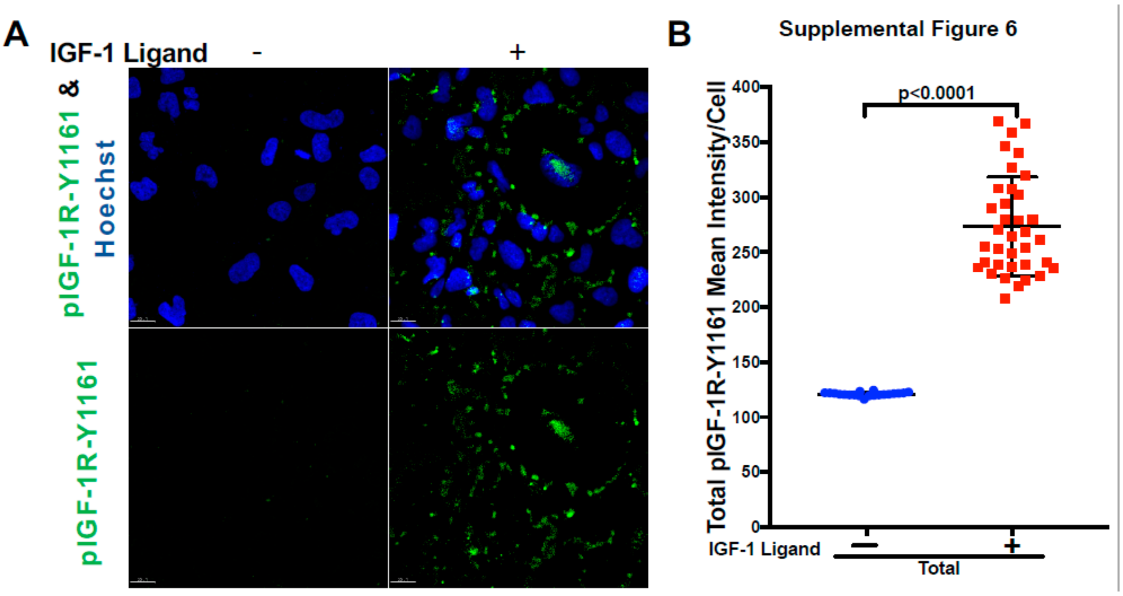

Figure S6. In vitro studies demonstrating that pIGF-1R-Y1161 is activated in A673 ES cells after 2 h of IGF-1 ligand stimulation. (**A)** Confocal microscopy images of A673 ES cells treated or not with 50 ng/ml of human IGF1 ligand for 2 hours. 20µm squares are shown. (**B)** Total pIGF-1R-Y1161 quantification in A673 ES cells treated or not with IGF-1 ligand (Figure panel A) using Imaris software.

Table S1. Summary information for the five trials analyzed in the meta-analysis.

Research Team	Treatment	Scheduled Research PET/CT	Participants (N)	Early PET/CT (N)	Percent Completed	Mean	Median
Kurzrock et al.	IGF-1R mAbs	No	5	3	60.00%	8.7	8
Anderson et al.	IGF-1R mAbs	Yes	17	16	94.12%	14.2	13
Pappo et al.	IGF-1R mAbs	Yes	13	13	100.00%	9.2	8
Nang et al.	IGF-1R/mTORi	No	11	2	18.18%	10.5	10.5
Schwartz et al.	IGF-1R/mTORi	No	5	2	40.00%	7.5	7.5

Table S2. *P*-values associated with early CT and PET imaging.

Early imaging response (day 7–14)	Response	Clinical Benefit	PFS	OS
WHO (CT imaging)	0.0014	0.0002	0.0001	0.0004
RECIST (CT imaging)	0.0011	0.0001	0.0012	0.0075
EORTC (PET imaging)	0.0044	0.0026	0.0418	0.0063
PERCIST (PET imaging)	0.0049	0.0051	0.0543	0.0058
TLG (PET imaging)	0.0002	0.0001	0.0008	0.0112

p-values for each of the early PET/CT measures of tumor response, obtained between 7–26 days after starting IGF-1R or IGF-1R/mTOR-based therapy, with respect to percent clinical response, clinical benefit, PFS, and OS.

Table S3. Receiver Operating Characteristics.

Early imaging response (median 10-days post treatment)	*p*-value	ROC (AUC)
WHO (CT imaging)	0.2286	0.7
RECIST (CT imaging)	0.4745	0.6
EORTC (PET imaging)	0.2700	0.65
PERCIST (PET imaging)	0.2039	0.69
TLG (PET imaging)	0.1173	0.74

Logistic regression to determine complete or partial response vs. stable disease among patients that achieved clinical benefit. Among 5 variables considered total lesion glycolysis (TLG) demonstrated the best performance.

Table S4. Individual patient data for responders and non-responders.

**Patient Data**						**Response**		**IHC**	**pIGF1R Staining**	
**RECIST Response**	Patient	Treatment Type	Best RECIST Response	PFS (Months)	OS (Months)	Avg PFS (Median)	Avg OS (Median)	pIGF-1R Positive	Avg PFS (Median)	Avg OS (Median)
Response	Responder 1	IGF-1R/mTOR	CR	37.3	65.5	12.4 (8.45)	46.78 (41)	No	12.68 (9.7)	46.2 (47.3)
	Responder 2	IGF-1R	PR	4.5	17.2			No		
	Responder 3	IGF-1R	PR	2.8	18.6			No		
	Responder 4	IGF-1R	PR	7.7	33			No		
	Responder 5	IGF-1R/mTORi	PR	12.1	61.5			No		
	Responder 6	IGF-1R	PR	11.7	81.6			No		
	Responder 7	IGF-1R	PR	26.4	100.9			Yes	4.88 (1.45)	20.2 (6.5)
	Responder 8	IGF-1R	PR	9.2	33.6			Yes		

	Responder 9	IGF-1R	PR	5.3	6.7			Yes
	Responder 10	IGF-1R/mTORi	PR	7	49.2			Yes
No Response	Non-Responder 1	IGF-1R	PD	1.4	5.6	1.33 (1.45)	6.55 (5.4)	Yes
	Non-Responder 2	IGF-1R	PD	1.3	6.3			Yes
	Non-Responder 3	IGF-1R	PD	1.4	5			Yes
	Non-Responder 4	IGF-1R	PD	1.5	2.7			Yes
	Non-Responder 5	IGF-1R	PD	1.1	10.2			Yes
	Non-Responder 6	IGF-1R	PD	1.4	16.3			Yes
	Non-Responder 7	IGF-1R/mTORi	PD	0.7	1.1			Yes
	Non-Responder 8	IGF-1R/mTORi	SD	1.8	5.2			Yes

Clinical characteristics of each of the patients for which pre-treated IHC markers were available for analysis.

© 2020 by the authors. Licensee MDPI, Basel, Switzerland. This article is an open access article distributed under the terms and conditions of the Creative Commons Attribution (CC BY) license (http://creativecommons.org/licenses/by/4.0/).
